# The high-intensity reflectometer of the Jülich Centre for Neutron Science: MARIA^1^

**DOI:** 10.1107/S1600576718006994

**Published:** 2018-05-29

**Authors:** Stefan Mattauch, Alexandros Koutsioubas, Ulrich Rücker, Denis Korolkov, Vicenzo Fracassi, Jos Daemen, Ralf Schmitz, Klaus Bussmann, Frank Suxdorf, Michael Wagener, Peter Kämmerling, Harald Kleines, Lydia Fleischhauer-Fuß, Manfred Bednareck, Vladimir Ossoviy, Andreas Nebel, Peter Stronciwilk, Simon Staringer, Marko Gödel, Alfred Richter, Harald Kusche, Thomas Kohnke, Alexander Ioffe, Earl Babcock, Zahir Salhi, Thomas Bruckel

**Affiliations:** a Jülich Centre for Neutron Science JCNS, Forschungszentrum Jülich GmbH, MLZ, Lichtenbergstrasse 1, 85747 Garching, Germany; b Jülich Centre for Neutron Science JCNS and Peter Grünberg Institut PGI, JARA-FIT, Forschungszentrum Jülich GmbH, 52425 Jülich, Germany; c ZEA-1, Forschungszentrum Jülich GmbH, 52425 Jülich, Germany; d ZEA-2, Forschungszentrum Jülich GmbH, 52425 Jülich, Germany; e G-ELI, Forschungszentrum Jülich GmbH, 52425 Jülich, Germany

**Keywords:** polarized neutron reflectometry, magnetism, grazing-incidence small-angle neutron scattering, GISANS

## Abstract

MARIA (magnetism reflectometer with high incident angle) is a world class vertical sample reflectometer dedicated to the investigation of thin films in the fields of magnetism, soft matter and biology. The elliptical vertically focusing guide allows one to measure small samples with a typical size of 1 × 1 cm very efficiently. The double-bounce polarizer and the *in situ* pumped ^3^He SEOP (spin-exchange optical pumping) neutron spin filter cell for analysing the polarization of the reflected neutron beam can be moved into the beam in seconds. The polarized flux of MARIA amounts to 5 × 10^7^ n (s cm^2^)^−1^ at the sample position with a horizontally collimated beam of 3 mrad, a wavelength of λ = 4.5 Å and a wavelength resolution of Δλ/λ = 10%. In the non-polarized mode a flux of 1.2 × 10^8^ n (s cm^2^)^−1^ is achieved in this configuration. MARIA is also capable of grazing-incidence small-angle neutron scattering measurements, using a pinhole collimation with two four-segment slits and an absorber that prevents the focusing of the elliptical guide in the vertical direction.

## Abstract 2

MARIA is a world class vertical sample reflectometer dedicated to the investigation of thin films in the fields of magnetism, soft matter and biology. With the elliptical vertically focusing guide and a wavelength resolution of Δλ/λ = 10%, the non-polarized flux at the sample position amounts to 1.2 × 10^8^ n (s cm^2^)^−1^. Besides the polarized and non-polarized reflectivity mode for specular and off-specular reflectivity measurements, MARIA can also be used to carry out grazing-incidence small-angle neutron scattering investigations.

## 1. Science case of MARIA   

MARIA (magnetism reflectometer with high incident angle) (Mattauch *et al.*, 2015 ▸) is a vertical sample reflectometer dedicated to the investigation of thin films and layered heterostructures. In recent years neutron reflectometry (NR) has emerged as a powerful tool for resolving structures at interfaces, with the ability to distinguish structural features normal to the interface with sub-nanometre resolution. The technique has attracted a large international user community covering a wide range of scientific disciplines in the areas of soft and hard matter. Techniques of unpolarized and polarized NR in combination with polarization analysis or appropriate deuteration labelling have enabled the internal structure of different components of complex systems to be resolved on a sub-nanometre length scale. For example, green solvents (Sirard *et al.*, 2003 ▸) as well as hybrid structures consisting of magnetic and non-magnetic components separated by interfaces (Decher & Schlendorf, 2012 ▸) and synthetic antiferromagnets with ferromagnetic layers periodically interleaved with metallic or insulating spacers, attractive for spintronic applications (Chen *et al.*, 2017 ▸), have been studied. One of the most important problems of hard matter physics of thin films is the investigation of the properties of interfaces, like superconductivity between insulating materials, induced magnetism between non-magnetic layers, ferromagnetism between antiferromagnetic layers *etc.* Furthermore, important research topics include interdiffusion mechanisms at the interface or the entire area of ordered lateral structures, whether they are magnetic or not.

In parallel, neutron reflectometry has proven to be quite useful in applications at the interface between physical chemistry and biology. An important ‘blind spot’ in the area of molecular biology is the structural biology of membrane proteins (Shenoy, Shekhar *et al.*, 2012 ▸; Shenoy, Nanda & Lösche, 2012 ▸; Nanda *et al.*, 2010 ▸; Datta *et al.*, 2011 ▸; Pfefferkorn *et al.*, 2012 ▸). Biomembranes are intrinsically disordered and many membrane proteins bear functionally important regions which are structurally disordered. More than 30% of the cell’s protein inventory are membrane incorporated or membrane associated (*i.e.* are membrane proteins). These are severely understudied in comparison to cytosolic (*i.e.* dissolved) proteins owing to difficulties related to their crystallization and complications in the application of standard experimental techniques that work in bulk solution. Neutron reflectometry on engineered membrane-mimetic interfaces (*i.e.* solid-supported bilayers) is a tremendously important future tool for the structural characterization of membrane proteins, identifying their association and localization in or on the lipid bilayer and their mutual interaction. Furthermore, the investigation of hybrid materials, with an interface between two different classes of materials, and the biomineralization and bio-compatibility of materials like implants are very important fields for the near future.

A scientific field which will profit from polarized grazing-incidence small-angle neutron scattering (GISANS) and off-specular scattering is the field of magnetic nanostructures. For the study of magnetic correlations, grazing-incidence scattering with neutrons is the method of choice, since neutrons directly measure the magnetic order parameter and thus provide model-free details on the magnetic structure. Applying polarization analysis, depth-resolved plane-perpendicular and lateral vector magnetometry can be performed on an absolute scale, unrivalled by any other technique. One example is magnetic nanoparticles assembled in a non-magnetic matrix of a thin film (Wang *et al.*, 2017 ▸). The structure can be stabilized by a polymer and the interaction between the particles can be tuned by adding magnetic nanoparticles (Lu *et al.*, 2007 ▸; Erb *et al.*, 2009 ▸). It is particularly interesting to study the interplay between the magnetization behaviour and magnetic ordering of the nanoparticles and the vertical and lateral structure of the particular matrix material (*e.g.* polymer). Here neutrons deliver contrast for both classes of materials (soft matter and magnetic) at the same time. The magnetic domain structure and multilayer structures in thin films can vary from a few nanometres up to several micrometres (Kentzinger *et al.*, 2007 ▸). Polarized GISANS allows access to the sub-micrometre scale, while the micrometre regime can be studied by polarized off-specular scattering (in reflectometry mode). Knowledge of the static domain behaviour and the in-plane and inter-plane correlation lengths of the domain structure (in their dependence on the external parameters) is crucial in understanding the underlying physics. As an example, the domain evolution in rare earth (RE) metals and alloys of RE and 3*d* transition metals are still insufficiently understood. Chirality effects found in Ho/Y and in Dy/Y multilayers and some other RE alloys with a preferred domain formation of left or right handed helixes (Grigoriev *et al.*, 2008 ▸) are of fundamental interest and the exploitation of chirality effects is one promising route for future spintronic applications.

All these effects in the hard matter, soft matter and biology communities can be combined under the title ‘understanding and controlling interfacial structures and interactions in the 10 nm regime’. MARIA is dedicated to these scientific fields.

## 2. Conclusion from the science case for the instrument layout   

**Figure 1:**
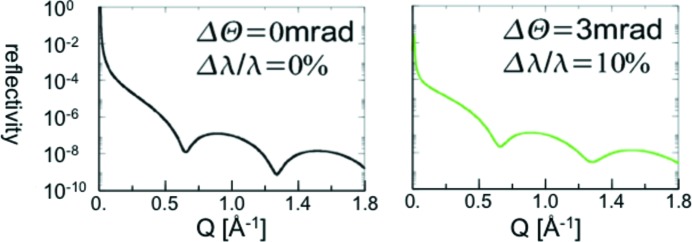
Simulated reflectivity curve of a 10 Å thick iron layer on top of an Si substrate with wavelength 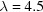
 Å. Left: perfect collimation and wavelength resolution. Right: relaxed collimation of 3 mrad and wavelength resolution of 10%. As there is nearly no difference visible, thin layers can be easily measured with a relaxed wavelength resolution without losing too much information.

From the science case we learned that the reflectometer is dealing mostly with thin interfaces with a low amount of scattering volume. To study these successfully one has to maximize the intensity on the sample as much as possible. In Fig. 1 ▸ the calculated reflectivity curve of a 10 Å thick Fe layer on top of an Si substrate is shown. Nearly no difference is visible between the left and right panel, with perfect collimation and wavelength resolution and with a relaxed collimation of 3 mrad and wavelength resolution of 10%, respectively. It is therefore obvious that thin layers can be investigated with relaxed wavelength resolution without losing much information, but gaining a huge factor in intensity. This even holds for thicker layers of up to 50 Å in the more commonly used 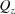 range of up to 0.25  Å^−1^. Furthermore, we can see from the simulated curve how important it is to cover a large dynamic range up to large *Q* values, as the first minimum in the reflectivity curve of the 10 Å thick Fe layer on an Si substrate can be found at 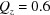
 Å^−1^ [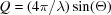, Θ is half the scattering angle and 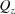 is the component of the scattering vector **Q** perpendicular to the layer]. It is desirable to reach the second minimum, which implies immediately a dynamic range larger than eight orders of magnitude. Therefore, MARIA has been designed to focus as many useful neutrons as possible on the sample position of a 1 cm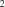 sample. To achieve this, MARIA is equipped with a velocity selector (VS) with a wavelength resolution of 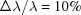 and with a vertically focusing guide.

## 3. Instrument layout   

### 3.1. Location   

**Figure 2:**
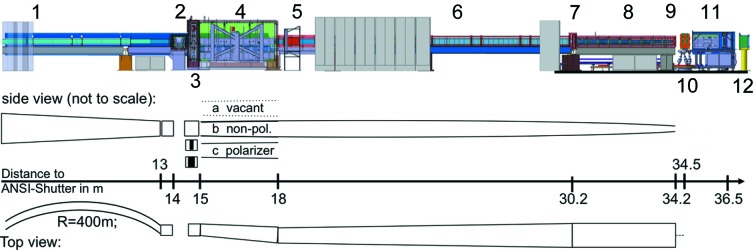
Side view of MARIA, starting on the left with (1) casemate wall, (2) VS, (3) lift with neutron guide and two Fermi chopper positions, (4) polarization chamber and lift with three positions, (5) radio frequency (RF) flipper, (6) elliptical vertically focusing NG (from 4 to 9), (7) slit S1, (8) collimation base, (9) slit S2, monitor 1 and attenuators, (10) hexapod with sample position and optional magnet, (11) detector arm with 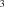
He filter and 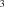
He two-dimensional position sensitive detector, and (12) beam stop.

MARIA is installed in the Neutron Guide Hall of the MLZ at the FRM II in Garching (Germany). In Fig. 2 ▸ a schematic side view of MARIA is shown. MARIA has been built at the end position of the neutron guide (NG) NL5-South, coming from the cold source, starting at the *Anlagen Sicherheits Verschluß* (ANSI-Shutter) with a guide cross section of 170 × 29 mm (height × width). The vertical height is compressed to 150 mm by a trumpet (*m* = 2 coating) along a length of 14 m in front of the velocity selector. Horizontally, the first 13 m are curved by a radius of 400 m, to separate the two neutron guides NL5-South and NL5-North from each other for geometrical reasons. Furthermore, the curvature together with the coating of the neutron guide reduces the white beam to a minimum wavelength 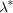 where 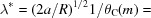 3.5 Å, with *a* = 29 mm, *R* = 400 m and 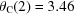
 mrad. Therefore, the number of neutrons that will be absorbed at the selector is strongly reduced. The last metre of the neutron guide in front of the VS is straight with a side wall coating of *m* = 2.

### 3.2. Wavelength and wavelength resolution   

**Figure 3:**
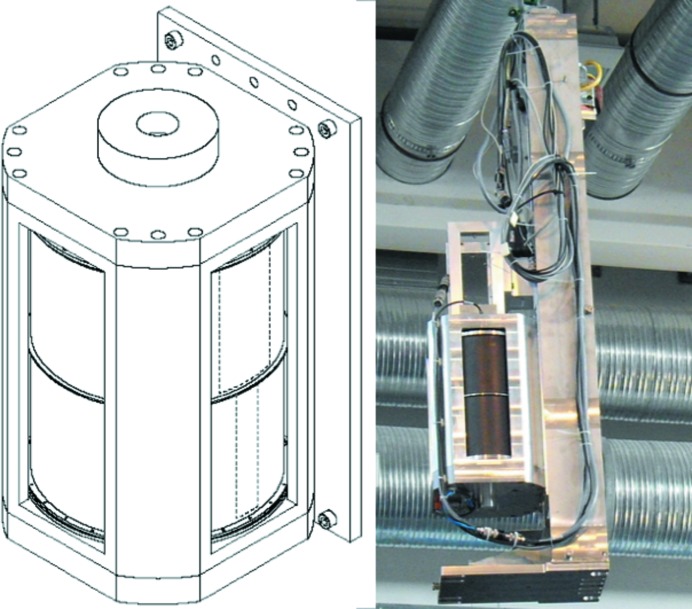
(Left) Sketch of the Fermi chopper at MARIA. The Fermi chopper is based on two aluminium barrels coated by 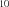
B in epoxy. The omitted coating is indicated by the dashed rectangular regions on the right side of the FC. The larger window is used for 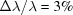 and the slimmer window for 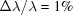. Both barrels are driven by a single motor. (Right) The Fermi chopper installed on the vertical lift, for changing between the neutron guide (on top of the Fermi chopper) and the two different wavelength resolutions.

The VS is a standard selector produced by ASTRIUM with the characteristic data of 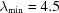
 Å at 28 300 r min^−1^, 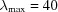
 Å at 3100 r min^−1^ and 72 absorbing blades with a 48.3° screw angle. The resulting wavelength resolution is 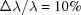. The entrance and exit windows are at the nine o’clock position and have a maximum height of 150 mm and a width of 29 mm, fully matching the cross section of the beam. The accessible λ ranges are separated in three intervals of [4.5–12.5 Å], [17–27 Å] and [36–40 Å]. A special feature of this VS is that the frame of the exit window is coated on the inner surface with a layer of 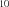
B
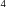
C epoxy to absorb the edges of the neutron beam before it can hit the aluminium housing and create hard prompt γ-radiation. The intensity of the monochromatic neutron beam is monitored by a 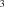
He finger detector which is mounted close to the exit of the VS. Behind the VS a lift with three positions is installed, where two positions are occupied by a Fermi chopper (FC) setup built by ASTRIUM (see Fig. 3 ▸) and one position with a neutron guide. Each position has a length of 0.75 m. The FC is used to achieve better wavelength resolution in the time-of-flight (TOF)-mode chopping wavelength band selected by the VS, creating neutron pulses 22 m in front of the detector. The barrel of the FC is made from aluminium and coated by a 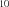
B layer on both sides. At the window positions (indicated by the dashed line in Fig. 3 ▸) the coating is omitted. The upper barrel has two windows with a cross section of 70 × 150 mm, resulting in a wavelength resolution of 3%, and the lower barrel has windows with a cross section of 30 × 150 mm, resulting in a 1% wavelength resolution in TOF mode. These two FCs for 1 and 3% wavelength resolution are placed into the middle and bottom positions. The upper position houses a straight NG and passes the 10% beam with minimal losses.

### 3.3. The polarizer and the elliptically focusing neutron guide   

**Figure 4:**
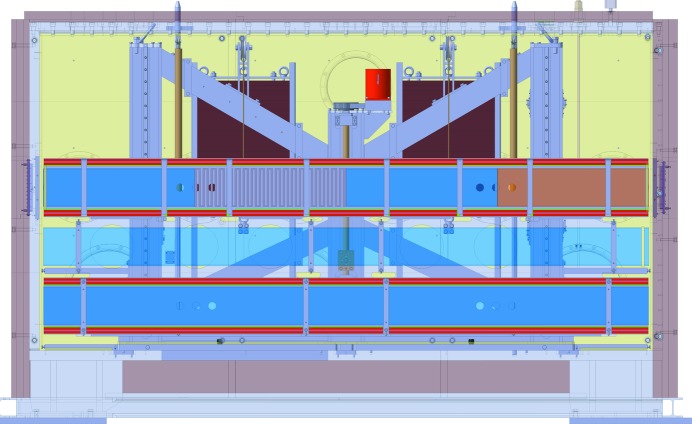
Side view of the polarization chamber with the lift and the three different guide positions. The lowest position is occupied by a polarizing NG. The red bars above and below the blue NG indicate the yoke filled with permanent magnets. The middle position is used by the unpolarized NG and the top position, which is in reality vacant, is in this technical drawing occupied by a tentative combination of a polarizing NG and a Drabkin wiggler. The two dark-red blocks in the background are counter weights to reduce the step motor load of the lift.

**Figure 5:**
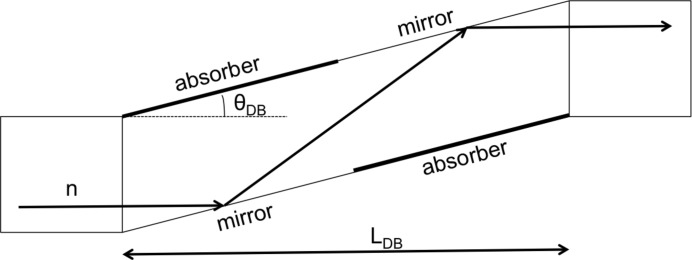
Top view of the double-bounce mirror used at MARIA for polarizing the neutron beam (sketch not to scale).

**Figure 6:**
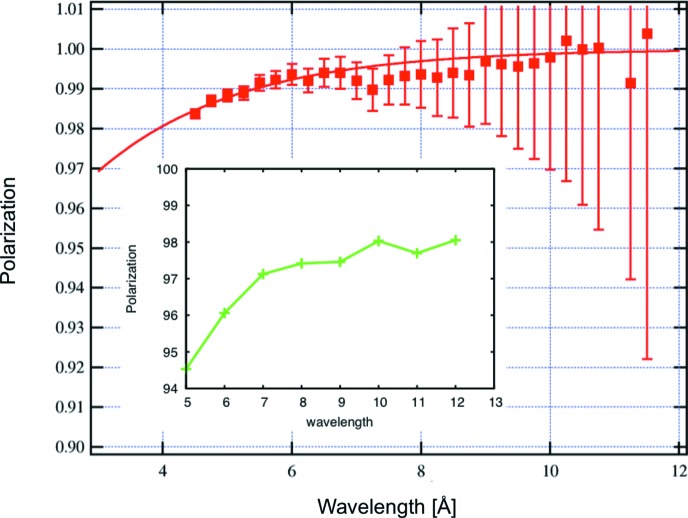
Measurement points of the efficiency of the polarizer, neglecting the adiabatic RF flipper inefficiency and the 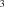
He spin-exchange optical pumping (SEOP) filter efficiency, with an exponential guide to the eye. The performance is very good at 98.3% at the minimum wavelength of 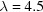
 Å and approaches 100% for wavelengths around 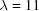
 Å. The inset shows the analysing power of the 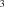
He SEOP neutron spin filter with 94.5% polarization at 5 Å and 98% polarization at 12 Å.

**Figure 7:**
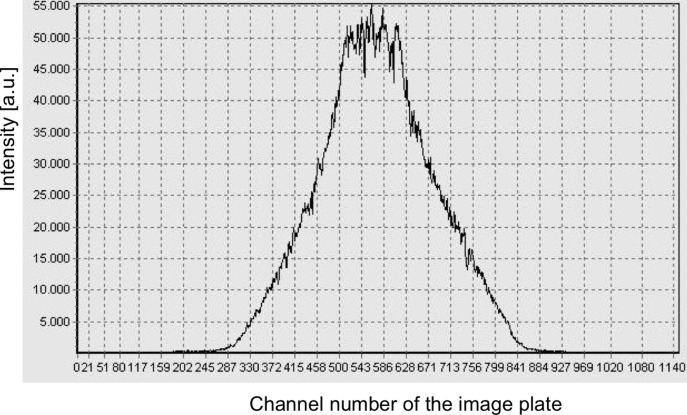
The vertical beam profile of the elliptical vertically focusing neutron guide at the sample position. The plateau of the focused beam is clearly seen and has a height of 106 channels, amounting to 21 mm as the imaging plate is binned to 200 µm per channel.

Downstream of the chopper chamber the housing of the polarizer is attached, containing in the same way a three-level lift which allows users to switch between a polarizing NG (bottom position) and an unpolarized NG (middle position) within seconds (Fig. 4 ▸). The length of the NG inside the housing is 3020 mm. Currently the third and top position is vacant, allowing for future instrument development. The elliptically focusing neutron guide in the vertical direction starts in this section. The shape of the truly elliptically curved guide is given in Table 1 ▸. In the horizontal direction the NG has a double-bounce kink as depicted in Fig. 5 ▸. The double kink is inclined by an angle 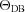 = 1.1°, covering in this way with the first mirror the width of the neutron guide (29 mm) and leading to a total length of 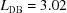
 m. In this way beams in the wavelength range between 4.5 and 12 Å are fully polarized by using a coating of *m* = 3.5 reflecting polarizing supermirror (FeSi). For the non-polarizing section the same geometry and the same coating of Ni–Ti supermirrors is used. Downstream of the polarizer an RF flipper is installed. The polarizing efficiency of the double kink and the RF flipper is shown in Fig. 6 ▸. Behind the polarizer, the guide starts to widen in the horizontal direction linearly from 29 to 40 mm over a length of 12.2 m up to slit S1 at the beginning of the collimation. The left/right (l/r) coating of the NG elements has *m* = 1, while the top/bottom (t/b) coating is still *m* = 2 from the start of the polarizer down to the collimation. The collimation itself has a length of 4 m and is accompanied by two four-segment slits: S1 with a cross section of maximum 148.8 × 40 mm (h × w) and S2 with a cross section of maximum 50 × 50 mm (h × w). The shape of the NG forms a C, with a top/bottom coating of *m* = 4, a right coating of *m* = 0 (absorber) and the left side open. In the reflectometry mode the top and bottom segments of slits S1 and S2 are fully open, allowing the full vertically focused beam to be incident on the sample. In Fig. 7 ▸ a vertical cut of the beam profile is shown, measured at the sample position with an imaging plate. The focusing effect is clearly seen and the plateau has a width of 21 mm.

### 3.4. GISANS mode   

**Figure 8:**
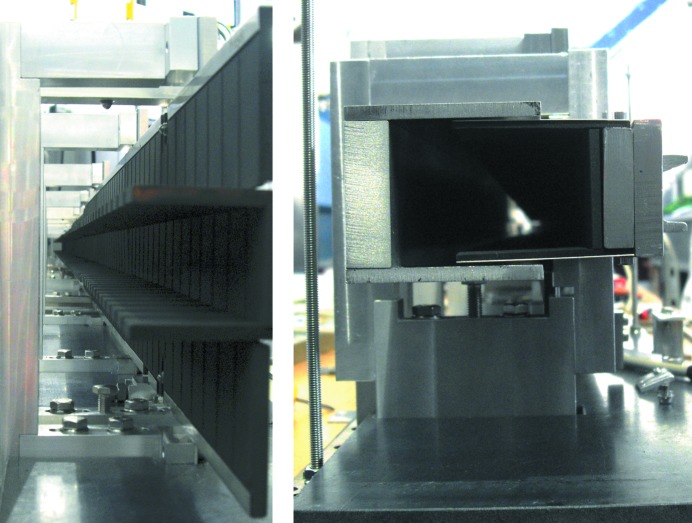
(Left) Vertical and horizontal absorber plates of B_4_C, designed to avoid the focusing in the GISANS mode of MARIA. (Right) The absorber plates are moved into the vertical elliptical NG and form together with the slits S1 and S2 a tunnel for the double-collimated neutron beam.

In the GISANS mode the absorbing insert shown in Fig. 8 ▸ is moved over the full length of the collimation into the NG, forming together with the slits S1 and S2 a pin hole geometry resulting in a double-collimated beam. In this mode the maximum vertical opening of S1 and S2 is 48 mm. The full flight path starting downstream of the VS up to the end of the collimation is kept under vacuum without any additional window. Between the vacuum window at the exit of the collimation (made of 4 mm thick double-polished single-crystal sapphire) and the slit S2, a set of three attenuators from 2 mm thick Borofloat33 glass from Schott, can individually be moved into the beam, where each single attenuator reduces the beam intensity by one order of magnitude for a wavelength of 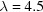
 Å. Furthermore, a 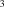
He finger detector is installed to monitor the beam in front of S2. Behind S2 an evacuated flight path of length 300 mm can be installed optionally to reduce air scattering.

### 3.5. Sample area and detector arm   

**Figure 9:**
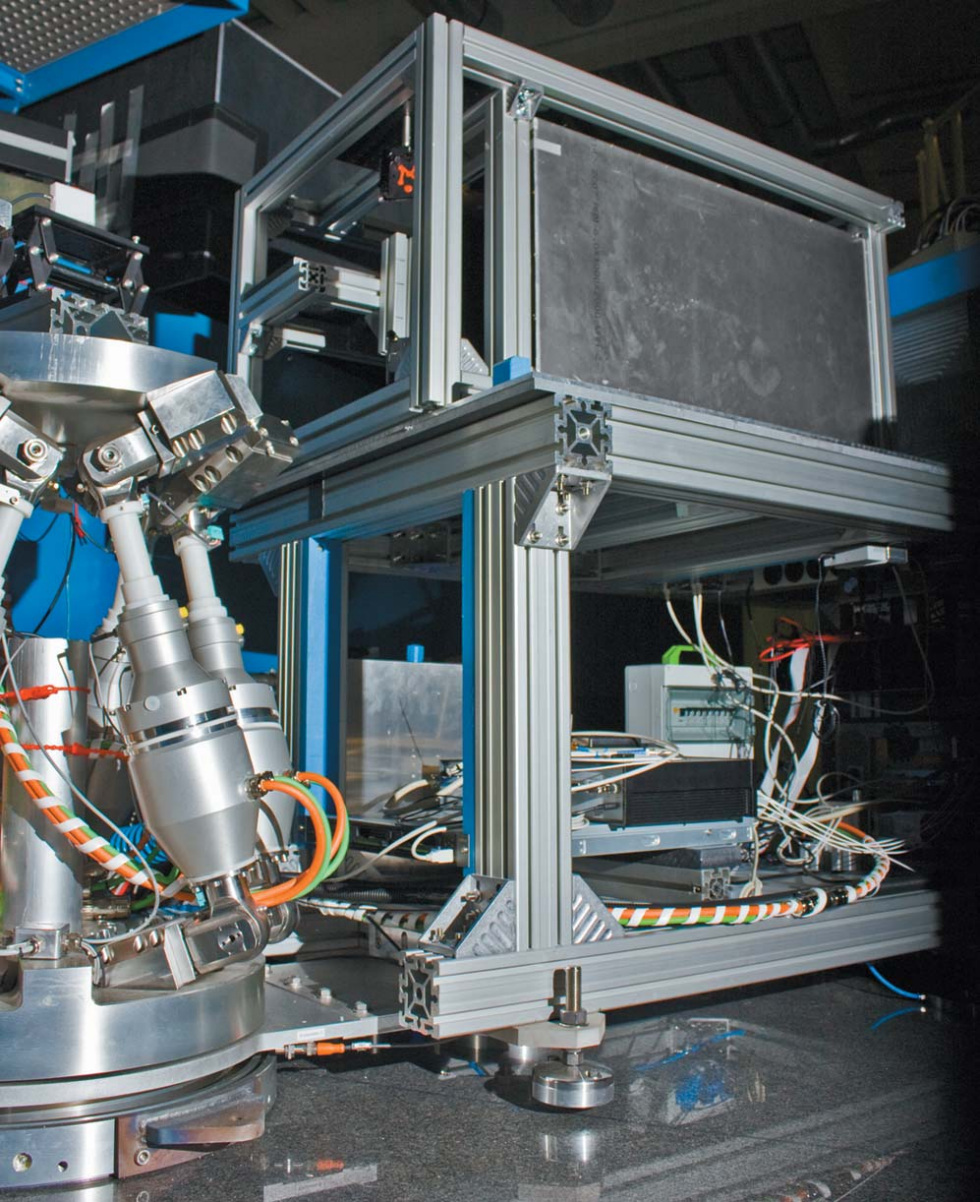
View of the open detector arm with the 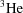 SEOP wide-angle spin filter installed and a glimpse on the hexapod at the sample position.

The sample table, a hexapod (Ohe, Switzerland), is located 50 cm behind the end of the ellipse and can carry a load of 550 kg, keeping an accuracy of 1/1000 mm in translations and 1/100° for rotations. In contrast to a classical combination of linear stages and cradles, the hexapod can rotate the sample around a virtual centre which is not restricted by any means. Furthermore, the movement of the six legs of the hexapod is done simultaneously with a fully electrically controlled speed, so that a constant rotation or translation speed of the sample can be set, including a starting and stopping ramp. This feature is used for kinetic measurements (see §6) with the rotation of the ω axis. The hexapod movements are given in a Cartesian frame and not in the frame of the six legs. Additional to the six axes of the legs, the sample table has one more rotation axis and one friction wheel. The rotation axis is parallel to the ω axis and allows rotation of the entire hexapod by 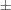
180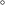. The friction wheel can move the detector arm (see Fig. 9 ▸) between −7 and 100°, on which an *in situ* pumped SEOP 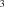
He wide-angle spin filter (Babcock *et al.*, 2011 ▸; Salhi *et al.*, 2014 ▸) with a diameter of 12 cm is installed as close as possible to the sample. With a distance of 650 mm to the sample the cell covers 90% of the detector width and height. Within seconds the wide-angle spin filter can be moved horizontally out of the beam for a pure unpolarized measurement. Last but not least, at the end of the instrument (2 m downstream of the sample) a detector together with two beam stops of 6 mm thick 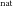
B
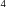
C for the GISANS (square shape: 60 × 40 mm) and the reflectometry mode (rectangular shape: 60 × 400 mm) are installed. Both beam stops can be moved over the entire width of the detector and rotated around their axes to adapt the covered area. The detector is a 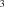
He delay line two-dimensional position sensitive detector from DENEX and has a size of 400 × 400 mm with a spatial resolution of 2 × 3 mm (h × v). The detector efficiency for 4 Å neutrons is better than 60% and the maximum global count rate is ∼10^6^ n s^−1^. The entire detector arm is under Ar atmosphere to reduce the background from air scattering. To cut the direct beam as early as possible, an additional beam stop is installed in front of the detector housing.

In this way MARIA is achieving a very good performance and is one of the most intense neutron reflectometers in the world. With gold foil measurements at the sample position we have measured an intensity of 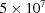
 n (s cm^2^)^−1^ in the polarized mode for a 3 mrad collimated beam for 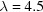
 Å and 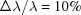. In the non-polarized mode a flux of 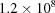 n (s cm^2^)^−1^ is reached in the same configuration.

## 4. Simulations   

**Figure 10:**
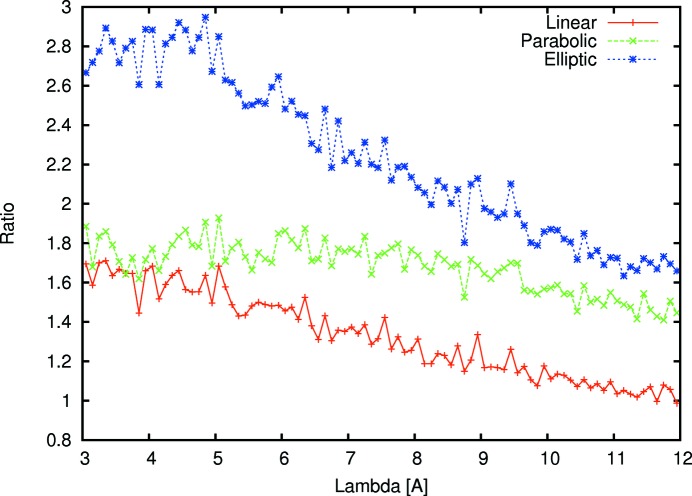
Gain comparison of elliptical, parabolic and linear focusing guides over a constant cross section NG for a 1 × 1 cm sample area half a metre behind the NG exit.

**Figure 11:**
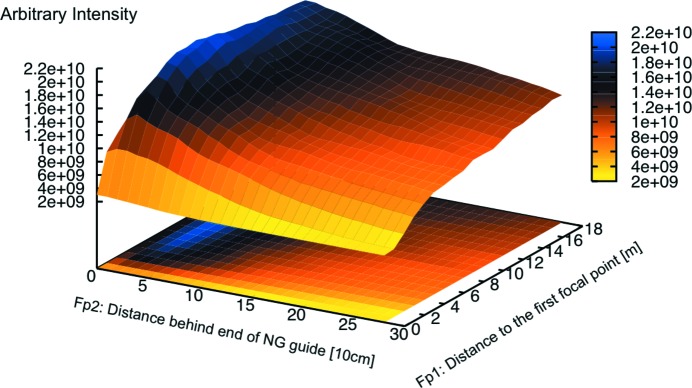
Systematic approach to find the best elliptical shape for focusing the neutrons on a 1 × 1 cm area on the sample position. fp1 is the distance from the first focal point of the ellipse to the beginning of the elliptical NG, and fp2 is the distance from the 2nd focal point to the end of the elliptical NG. The maximum intensity is found with fp2 = 40 cm behind the end of the elliptical NG (focal point 10 cm in front of the sample position) and fp1 around 8 m in front of the elliptical NG.

The entire instrument has been simulated with the Monte Carlo package *Vitess* (Zsigmond *et al.*, 2002 ▸) to determine which vertical focusing NG should be used and which *m* coating of the NG is required. We also considered the hybrid solution of a velocity selector plus a Fermi chopper for increasing the wavelength resolution in TOF mode, and the type of polarizer. For the vertical focusing NG four different options were checked: a constant cross section (so no focusing), a linear compressing trumpet, and the two focusing methods of a parabola and an ellipse focusing on the sample. Beside the focusing type there are additional constraints. In the case of MARIA these constraints are the expected typical sample size of 1 cm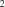 for thin films and heterostructures plus the distance of 0.5 m between the end of the guide and the sample position, which needs to allow enough space for a slit, attenuators, a monitor and the needed sample environment, like electromagnet, cryostat, cryo magnet, mobile molecular beam epitaxy (MBE) *etc.* These two restrictions immediately rule out the guide with a constant cross section and the linear trumpet. The trumpet increases the intensity at the exit of the NG quite heavily, but directly behind the guide the high diversion distributes the neutrons over a large area, so that the intensity on a 1 cm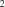 sample half a meter behind the guide is drastically reduced again. In the same way the small sample size demands a focus on the sample position. Fig. 10 ▸ shows the gain ratio of the elliptical focusing onto the sample, parabolic focusing onto the sample and the linear compressing NG *versus* a constant cross section NG on a 1 × 1 cm sample half a metre behind the NG exit. The elliptical, parabolic and linear NGs are the best of their class, starting in the polarization chamber with a 150 mm tall guide and having their focal point 19.73 m further downstream at the sample position. To find the best shape for the guides several different shapes were simulated and compared. In the case of the linear guide only the exit height can be varied, while in the case of the parabolic guide the focal point can be varied around the sample position. However in the case of the elliptical guide shape both focal points can be varied, the only constraint being that the neutron guide height at the start of the ellipse should match the height of 150 mm before. Taking a systematic approach to find the parameters of the elliptical NG, we simulated a two-dimensional map of the intensity with varying focal points fp1 and fp2 (see Fig. 11 ▸). The position for the maximum intensity is found with fp2 = 40 cm behind the end of the elliptical NG (focal point 10 cm in front of the sample position) and fp1 around 8 m in front of the elliptical NG. However, comparing the different gains in Fig. 10 ▸, we see that the elliptical shape has the best performance, focusing the neutrons over 0.5 m on an area of 1 × 1 cm.

## 5. The sample environment   

MARIA is equipped with a set of sample environments for the investigation of hard and soft matter samples. For hard matter, the most important devices are the standard electromagnet produced by Bruker Bio spin with four different sets of pole shoes, allowing a gap of 20 mm with a maximum field of 2.2 T, a gap of 50 mm with a maximum field of 1.2 T, a gap of 80 mm with 0.7 T or a gap of 100 mm with 0.5 T. The field is fully controllable from the instrument computer, allowing for various measurements starting from zero field cooling up to the saturation field of most materials. Additionally, a 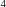
He closed-cycle cryostat can be installed inside the magnet, allowing for temperature-dependent measurements down to 3 K with the standard sample size of 20 × 20 mm. Moreover a sample holder for 2 inch (50.8 mm) diameter samples is available, but only in a reduced field of 0.75 T and at temperatures down to 5 K. We plan to build a special setup for small samples of around 10 × 10 mm which will fit into the small pole shoe gap of 20 mm and will provide a maximum field of 2 T down to 10 K. For investigations which require an electrical field applied to the sample under cooling inside the cryostat, a supply for voltages of up to 500 V is available.

Furthermore MARIA can use the compensated 5 T cryomagnet of the Jülich Centre for Neutron Science, allowing for measurements with the milliK inset down to 50 mK.

**Figure 12:**
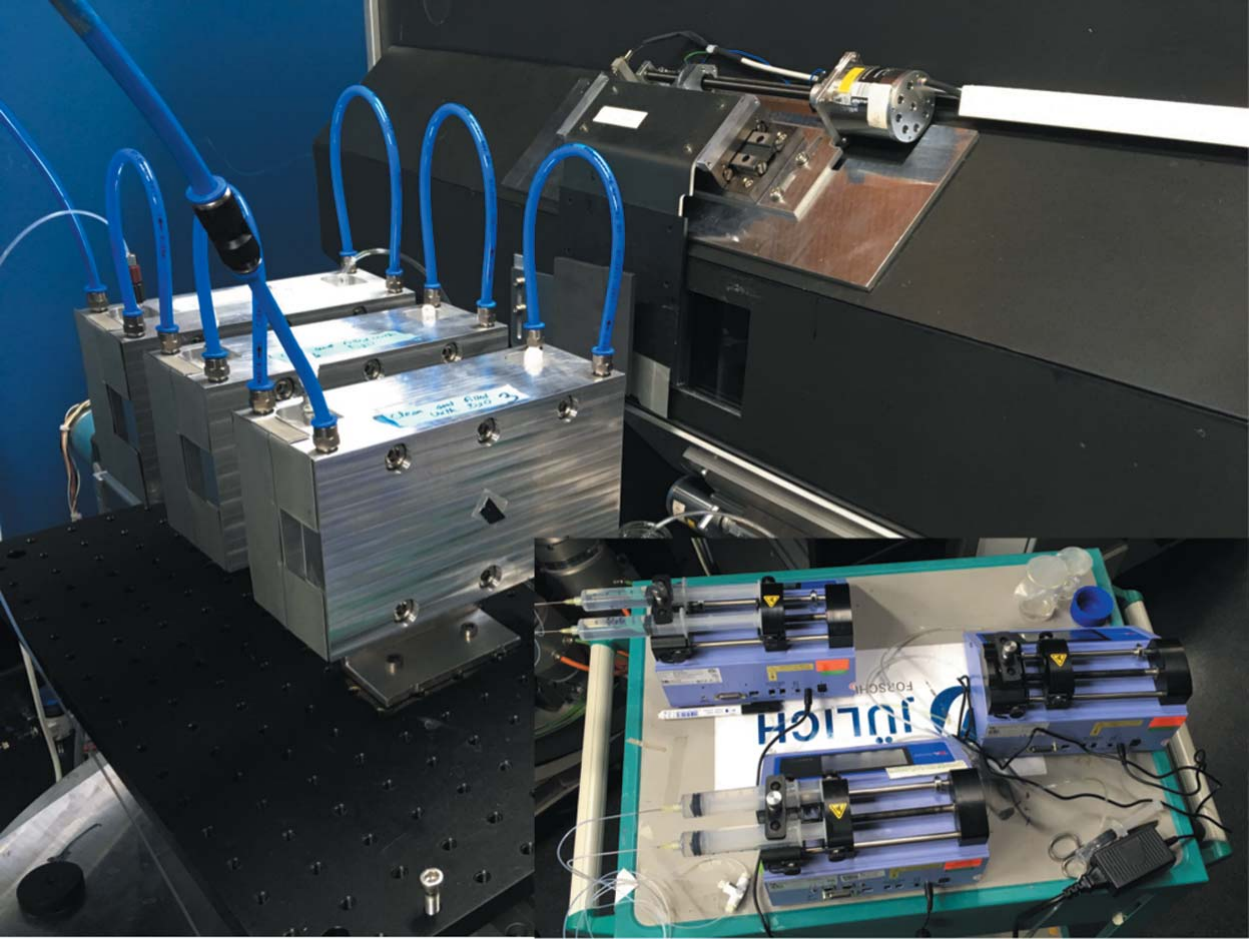
Three liquid cells on the sample changer at the sample position of MARIA connected to a Julabo (not visible) for temperature control. In the lower right corner three remotely controlled syringe pumps are shown, connected to the liquid cells in the main picture.

For the soft matter and biology communities, MARIA is equipped with two different types of temperature-controlled liquid cells which allow the investigation of liquid/solid interfaces (see Fig. 12 ▸). The smaller cell type has been built to accommodate standard 2 inch Si wafers of 5 mm thickness and is oriented more towards reflectivity studies. The utilization of such a small surface wafer is made possible by the beam-focusing nature of the instrument. By utilizing the on-site molecular beam epitaxy infrastructure (see below) many different types of metal films can be grown. In this respect a magnetic alloy backing film may be deposited on the wafer’s surface and, in conjunction with the usage of the instrument’s magnet, the ability to controllably manipulate the contrast of the supporting film without the need for solvent exchange is provided. Such a feature can greatly aid users in resolving surface structures through elaborate data fitting (Majkrzak *et al.*, 2000 ▸). The larger cell accommodates long silicon blocks of 10 cm length, having in mind the need not to over-illuminate the wafer surface under grazing incidence, which is the standard condition during GISANS studies. Up to four liquid cells can be simultaneously mounted on the sample stage, and remotely controlled solvent exchange can be performed using connected syringe pumps. In general, for both cell types special care is taken in order to minimize background scattering from cell components.

Cells that have been custom made by the users and sample environments that are tailored to specific systems are welcome. Experience has shown that usually sample cells that have been fabricated for horizontal reflectometers can also be used on MARIA with minor modifications.

**Figure 13:**
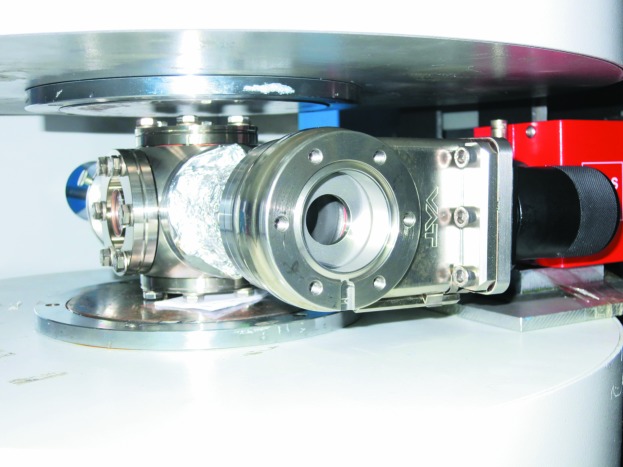
The versatile UHV transfer chamber that bridges the production of the sample inside the MBE system and the measurement at MARIA. The transfer chamber is placed inside the electromagnet and the exit window of the beam is at the centre of the photograph. On the left hand side is a window for the pre-alignment of the sample with a laser, and on the right hand side the red getter pump is visible.

The latest development is a versatile ultra-high-vacuum (UHV) transfer chamber (Mohd *et al.*, 2016 ▸) that bridges the production of the sample inside the MBE system [DCA M600 MBE system with a base pressure of 10
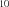
 mbar (1 bar = 100 kPa)] and the measurement at MARIA (see Fig. 13 ▸). The chamber is not only used for the transfer but additionally mounted on the instrument for the investigation of the sample, allowing for a maximum *Q* = 0.3 Å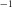 at 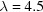
 Å. The base pressure in the transfer chamber is kept below 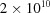
 mbar, resulting in a quasi *in situ* investigation. Using the transfer back and forth between MBE/MARIA it is possible to study the influence of the sample modification in the MBE system after an initial characterization at MARIA without any contamination by atmospheric gases in between.

## 6. Kinetic mode   

**Figure 14:**
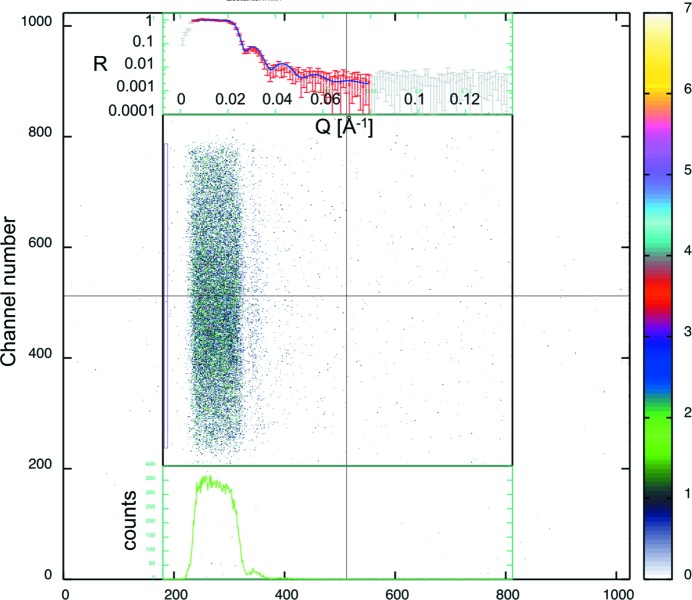
Detector image in kinematic mode of a measurement on an Ni supermirror with a layer thickness of *d* = 400 Å on a glass substrate.

**Figure 15:**
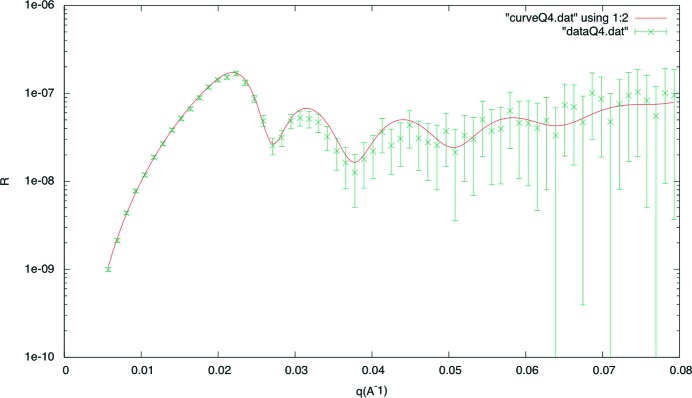
Fitted reflectivity curve plotted in 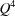 mode.

As already mentioned, the controller of the hexapod allows for a controlled rotation speed of the sample. This helps to speed up the measurement of a reflectivity curve, by placing the detector on a fixed position and turning the sample to sweep the reflectivity curve over the detector area. In this way, the positioning time and data collection time is saved, leading to short measurement times which are only dependent on the intensity of the incoming neutron beam and the background level. For a typical detector image in this mode, see Fig. 14 ▸. In the centre the raw detector image is shown, with the colour code on the right and the vertically integrated curve on a linear scale at the bottom. On top, the fitted reflectivity curve is plotted on a logarithmic scale, where the points used for the fit are plotted in red and the others plotted in grey. The blue solid line represents the fit including the background. For the measurement in Fig. 14 ▸ the horizontal opening of the slits S1 and S2 is set to 0.6 mm. The wavelength λ is set to 8.0 Å and the sample size is 2 × 4 cm (height × width). With a rotation speed of 2° s^−1^ for the sample, the measurement time for the fitted curve is 1.5 s. The fit result for the nominal 400 Å Ni layer on glass gives a thickness of *d* = 400.7 Å with a roughness of 7.5 Å and a scattering length density of the glass substrate of 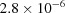
 Å^−2^. The scattering length density of Ni is kept constant at the literature value of 9.408 × 10
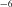
 Å^−1^. The quality of the fit is demonstrated by the plot in Fig. 15 ▸ where the measured points and the resulting curve of the fit are plotted against *Q* 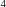. In this way, quick measurements in the time range of 1–2 s probing reflectivity curves in a dynamic range of up to three orders of magnitude are possible. This enables soft matter studies for understanding material transport to and from membranes (Wacklin, 2009 ▸) as well as adsorption processes in the drug (Gutberlet *et al.*, 2004 ▸) and more detailed investigations of hydrogen storage materials examining the deuterium gas absorption and desorption of catalysts (Fritzsche *et al.*, 2012 ▸).

## 7. Conclusion   

MARIA is a world class vertical sample reflectometer dedicated to the investigation of thin films and heterostructures in the fields of magnetism, soft matter and biology. The wavelength resolution of 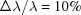 together with the elliptical vertically focusing guide lead to a polarized flux of 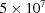
 n (s cm^2^)^−1^ at 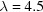
 Å with a horizontally collimated beam of 3 mrad. In the non-polarized mode this equals 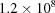 n (s cm^2^)^−1^ and allows one to measure small samples with a typical size of 1 × 1 cm very efficiently. For a polarized beam, the double-bounce polarizer can be moved into the beam in seconds and in the same way the *in situ* pumped 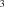
He neutron spin filter cell for analysing the reflected neutron beam is available. The sample environment covers the needs of a range of users, from a standard electromagnet in combination with the He closed cycle cryostat or the transfer chamber, to the 5 T cryo magnet. In the case of soft matter and biological samples, several cells are ready for use combined with the four-stage sample changer and remotely controlled syringe pumps. All this can easily be combined with both reflectometry modes (general step scan mode or fast kinetic mode) or with the GISANS mode. Switching between these modes is done in a few seconds and is possible *via* remote operation. The most important characteristics are summarized in Table 2 ▸ and typical applications of the MARIA instrument for the study of hard and soft matter systems can be found in a series of recent publications (Chen *et al.*, 2017 ▸; Strauß *et al.*, 2016 ▸; Koutsioubas, 2016 ▸; Jaksch *et al.*, 2015 ▸).

## Figures and Tables

**Table 1:** Sections of the reflectometer MARIA with the curvatures and coatings (abs. = absent)

Description	Length (m)	Radius (m)	Entrance (w × h) (mm)	Exit (w × h) (mm)	Coating b/t/l/r (m)
ANSI-Shutter	13	400	29 × 170.0	29 × 151.4	2.0/2.0/2.0/1.2
Up to selector	1	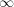	29 × 151.4	29 × 150.0	2.0/2.0/2.0/1.2
VS (gap)	0.3				
FC	0.75	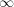	29 × 150.0	29 × 150.0	2.0/2.0/1.2/1.2
Polarizer	1.51	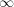	29 × 150.0	29 × 166.6	2.0/2.0/3.5/abs.
Polarizer	1.51	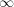	29 × 166.6	29 × 177.8	2.0/2.0/abs./3.5
Up to collimation	12.21	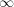	29 × 177.8	40 × 145.6	2.0/2.0/1.0/1.0
Collimation	4.0	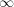	40 × 145.6	50 × 90.2	4.0/4.0/no guide/abs.
Sample position	0.5	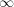			
Detector	2.0	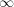			

**Table 2:** Characteristic data of MARIA

Scattering plane	Horizontal
Monochromators	VS + optional FC
λ range unpolarized	4.5–40 Å
λ range polarized	4.5–12 Å
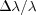	1% (VS + FC), 3% (VS + FC), 10% (VS)
Polarized flux	5 × 10 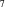 n (s cm^2^)^−1^ (3 mrad collimation)
Detector size	400 × 400 mm
Detector resolution	3 × 2 mm (h × v)
Sample–detector distance	1950 mm
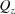 range	0.002–2.1 Å 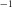
Detector angle ( 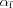 )	−7 to 100°
Polarization	Double-reflection polarizer (FeSi)
Polarization analysis	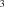 He SEOP filter pumped *in situ*
Collimation (scattering plane)	4 m long, slits 0–40 mm
Focusing	Vertically focusing elliptical NG
GISANS option	4 m long collimation
Q 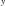 range	0.002–0.15 Å 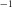

## References

[bb1] Babcock, E., Mattauch, S. & Ioffe, A. (2011). *Nucl. Instrum. Methods Phys. Res. A*, **625**, 43–46.

[bb2] Chen, B., Xu, H., Ma, C., Mattauch, S., Lan, D., Jin, F., Guo, Z., Wan, S., Chen, P., Gao, G., Chen, F., Su, Y. & Wu, W. (2017). *Science*, **357**, 191–194. 10.1126/science.aak9717 28706069

[bb3] Datta, S., Heinrich, F., Raghunandan, S., Krueger, S., Curtis, J., Rein, A. & Nanda, H. (2011). *J. Mol. Biol.* **406**, 205–214. 10.1016/j.jmb.2010.11.051 PMC3046808 21134384

[bb4] Decher, G. & Schlendorf, J. B. (2012). *Multilayer Thin Films: Sequential Assembly of Nanocomposite Materials*, 2nd ed. Weinheim: Wiley-VCH.

[bb5] Erb, R., Son, H., Samanta, B., Rotello, V. & Yellen, B. (2009). *Nature*, **457**, 999–1002. 10.1038/nature07766 19225522

[bb6] Fritzsche, H., Kalisvaart, W. P., Zahiri, B., Flacau, R. & Mitlin, D. (2012). *Int. J. Hydrogen Energy*, **37**, 3540–3547.

[bb7] Grigoriev, S. V., Chetverikov, Yu. O., Lott, D. & Schreyer, A. (2008). *Phys. Rev. Lett.* **100**, 197203. 10.1103/PhysRevLett.100.197203 18518483

[bb8] Gutberlet, T., Steitz, R., Fragneto, G. & Klösgen, B. (2004). *J. Phys. Condens. Matter*, **16**, S2469.

[bb9] Jaksch, S., Lipfert, F., Koutsioubas, A., Mattauch, S., Holderer, O., Ivanova, O., Frielinghaus, H., Hertrich, S., Fischer, S. F. & Nickel, B. (2015). *Phys. Rev. E*, **91**, 022716. 10.1103/PhysRevE.91.022716 25768540

[bb10] Kentzinger, E., Frielinghaus, H., Rücker, U., Ioffe, A., Richter, D. & Brückel, Th. (2007). *Physica B*, **397**, 43–46.

[bb11] Koutsioubas, A. (2016). *J. Phys. Chem. B*, **120**, 11474–11483. 10.1021/acs.jpcb.6b05433 27748120

[bb12] Lu, A.-H., Salabas, E. L. & Schüth, F. (2007). *Angew. Chem. Int. Ed.* **46**, 1222–1244. 10.1002/anie.200602866 17278160

[bb13] Majkrzak, C. F., Berk, N. F., Krueger, S., Dura, J. A., Tarek, M., Tobias, D., Silin, V., Meuse, C. W., Woodward, J. & Plant, A. L. (2000). *Biophys. J.* **79**, 3330–3340. 10.1016/S0006-3495(00)76564-7 PMC1301206 11106635

[bb14] Mattauch, S., Koutsioubas, A. & Putter, S. (2015). *J. Large-Scale Res. Facil.* **1**, 1–3.

[bb15] Mohd, A. S., Pütter, S., Mattauch, S., Koutsioubas, A., Schneider, H., Weber, A. & Brückel, T. (2016). *Rev. Sci. Instrum.* **87**, 123909. 10.1063/1.4972993 28040933

[bb16] Nanda, H., Datta, S. A., Heinrich, F., Lösche, M., Rein, A., Krueger, S. & Curtis, J. E. (2010). *Biophys. J.* **99**, 2516–2524. 10.1016/j.bpj.2010.07.062 PMC2955346 20959092

[bb17] Pfefferkorn, C. A., Heinrich, F., Sodt, A., Maltsev, A., Pastor, R. & Lee, J. (2012). *Biophys. J.* **102**, 613–621. 10.1016/j.bpj.2011.12.051 PMC3274814 22325285

[bb18] Salhi, Z., Babcock, E., Pistel, P. & Ioffe, A. (2014). *J. Phys. Conf. Ser.* **528**, 012015.

[bb19] Shenoy, S. S., Nanda, H. & Lösche, M. (2012). *J. Struct. Biol.* **180**, 394–408. 10.1016/j.jsb.2012.10.003 PMC3503488 23073177

[bb20] Shenoy, S. S., Shekhar, P., Heinrich, F., Daou, M.-C., Gericke, A., Ross, A. H. & Lösche, M. (2012). *PLoS One*, **7**, e32591. 10.1371/journal.pone.0032591 PMC3323581 22505997

[bb21] Sirard, S. M., Gupta, R. R., Russell, T. P., Watkins, J. J., Green, P. F. & Johnston, K. P. (2003). *Macromolecules*, **36**, 3365–3373.

[bb22] Strauß, F., Dörrer, L., Geue, T., Stahn, J., Koutsioubas, A., Mattauch, S. & Schmidt, H. (2016). *Phys. Rev. Lett.* **116**, 025901. 10.1103/PhysRevLett.116.025901 26824552

[bb23] Wacklin, H. P. (2009). *Biochemistry*, **48**, 5874–5881. 10.1021/bi802280b 19419199

[bb24] Wang, L.-M., Petracic, O., Kentzinger, E., Rucker, U., Schmitz, M., Wei, X.-K., Heggen, M. & Bruckel, Th. (2017). *Nanoscale*, **9**, 12957–12962. 10.1039/c7nr05097f 28831490

[bb25] Zsigmond, G., Lieutenant, K. & Mezei, F. (2002). *Neutron News*, **13**(4), 11–14.

